# Geographic Variation in the Petiole–Lamina Relationship of 325 Eastern Qinghai–Tibetan Woody Species: Analysis in Three Dimensions

**DOI:** 10.3389/fpls.2021.748125

**Published:** 2021-10-28

**Authors:** Yanan Li, Xiaomei Kang, Jieyang Zhou, Zhigang Zhao, Shiting Zhang, Haiyan Bu, Wei Qi

**Affiliations:** State Key Laboratory of Grassland Agro-ecosystems, School of Life Sciences, Lanzhou University, Lanzhou, China

**Keywords:** allometric relationship, altitudinal gradient, environmental stress, functional tradeoff, petiole cross-sectional area, resource allocation

## Abstract

The petiole–lamina relationship is central to the functional tradeoff between photosynthetic efficiency and the support/protection cost. Understanding environmental gradients in the relationship and its underlying mechanisms remains a critical challenge for ecologists. We investigated the possible scaling of the petiole–lamina relationships in three dimensions, i.e., petiole length (PL) vs. lamina length (LL), petiole cross sectional area (PCA) vs. lamina area (LA), and petiole mass (PM) vs. lamina mass (LM), for 325 Qinghai–Tibetan woody species, and examined their relation to leaf form, altitude, climate, and vegetation types. Both crossspecies analysis and meta-analysis showed significantly isometric, negatively allometric, and positively allometric scaling of the petiole–lamina relationships in the length, area, and mass dimensions, respectively, reflecting an equal, slower, and faster variation in the petiole than in the lamina in these trait dimensions. Along altitudinal gradients, the effect size of the petiole–lamina relationship decreased in the length and mass dimensions but increased in the area dimension, suggesting the importance of enhancing leaf light-interception and nutrient transport efficiency in the warm zones in petiole development, but enhancing leaf support/protection in the cold zones. The significant additional influences of LA, LM, and LA were observed on the PL–LL, PCA–LA, and PM–LM relationships, respectively, implying that the single-dimension petiole trait is affected simultaneously by multidimensional lamina traits. Relative to simple-leaved species, the presence of petiolule in compound-leaved species can increase both leaf light interception and static gravity loads or dynamic drag forces on the petiole, leading to lower dependence of PL variation on LL variation, but higher biomass allocation to the petiole. Our study highlights the need for multidimension analyses of the petiole–lamina relationships and illustrates the importance of plant functional tradeoffs and the change in the tradeoffs along environmental gradients in determining the relationships.

## Introduction

A complete leaf consists mainly of lamina and petiole. Lamina is the main functional structure for conducting photosynthesis to fix carbon. For most laminas, enhancing light interception efficiency, which depends partly on their petiole, is one of the key evolutionary mechanisms to improve photosynthesis (Niklas, 1999; Falster and Westoby, 2003; Niinemets et al., 2004; Perez et al., 2018). In addition, the petioles can serve multiple other functions, such as being a conduit for the transport of nutrients and sap (Yamada et al., 1999), providing mechanical support for the lamina (Niklas, 1992; Yamada et al., 1999), and adjusting leaf angle or leaf orientation to adapt to the variation in environments (Falster and Westoby, 2003). Most studies to date have indicated that the petiole–lamina relationship is fundamental to the functional tradeoff, e.g., the tradeoff between carbon gain and support costs, in most plant species (Niinemets, 1998; Pickup et al., 2005; Niinemets et al., 2006, 2007; Li et al., 2008; Zhong et al., 2019).

In woody communities, especially closed ones, self-shading among leaves reduces the efficiency of light interception by the lamina (Niinemets, 1998; Bell and Galloway, 2007; Roig-Villanova and Martíinez-Garcíia, 2016; Perez et al., 2018). Petiole elongation, at the expense of support, is one of the most obvious changes to diminish self-shading because it can send lamina to a higher position as well as adjust the angle of the lamina on a branch to avoid overlapping with its neighbors (King and Maindonald, 1999; Falster and Westoby, 2003; Bell and Galloway, 2007; Poorter and Rozendaal, 2008; Sarlikioti et al., 2011; Perez et al., 2018; Li et al., 2019; Zhong et al., 2019). The relationship between lamina length (LL) and petiole length (PL) is expected to be positive because of a higher proportion of overlap of longer laminas in a given space, which has been under genetic control (Tsukaya et al., 2002; Tsukaya, 2005). The positive relationship may be isometric or allometric, whereby the former can reflect an overall balance between adaptation to light interception vs. support costs in petiole growth, and the latter indicates a shift in the balance favoring LL. However, up to now, the relationship has not been examined in natural multispecies ecosystems, except for a few studies that found a positive correlation between PL and lamina area (LA; Niinemets et al., 2006; Xu et al., 2009). Notably, LA is a comprehensive index reflecting the length, width, and shape of the lamina (King and Maindonald, 1999; Niinemets et al., 2007; Vogel, 2009; Lin et al., 2020), and thus, these studies cannot determine whether the correlation is indirect mainly through LL, or whether lamina width and/or lamina shape exert additional effects on petiole length.

In the two-dimensional area space, theoretically, petiole crosssectional area (PCA) increases proportionally with increasing LA because a large total crosssectional area of vascular conduits can meet the high demand of photosynthesis or respiration for large laminas (as seen in the “pipe-model theory”; Niklas, 1992; Li et al., 2008; Ray and Jones, 2018). However, the petiole is also composed of epidermis and cortex for protection, support, and storage of nutrients. Thus, PCA is actually a measurement of “petiole structure,” representing the number and proportion of different tissues, e.g., vascular, storage, protective, or supportive. The proportion of these tissues in the petiole, however, is usually not fixed and varies significantly among species or environments (Givnish, 2002; Al-Edany and Al-Saadi, 2012; Gebauer et al., 2016; Maiti et al., 2016; Ray and Jones, 2018), potentially resulting in an interspecific allometric relationship between PCA and LA, whereby a larger variation in PCA than LA may attribute to a higher proportion of storage, protective, or supportive tissues in the petiole as the LA increases (Niinemets and Fleck, 2002; Klepsch et al., 2016; Filartiga et al., 2021; Sargin, 2021). Due to a significant positive relationship between lamina mass (LM) and LA (Pan et al., 2013; Lin et al., 2020), the PCA–LM relationship has been reported to be positive in some recent studies (Yamada et al., 1999; Levionnois et al., 2020). However, the mass of laminas with the equal area may be significantly different due to the differences in lamina structure (number of cell layers, the proportion of palisade cells, having or not protective tissue in leaf epidermis, etc. (Niklas, 1999; Niinemets and Fleck, 2002; Sack and Frole, 2006; Ray and Jones, 2018; Lin et al., 2020). As a result, LM may have an additional influence on the variation in PCA because, compared with small-mass leaves with the same LA, large-mass leaves need the petioles with larger PCA to meet the requirement for their leaf support and nutrition/water transport. Moreover, we expect high additional influence in the stressful subalpine/alpine environments where a higher proportion of protection tissue is required in the lamina.

Studies on biomass allocation have indicated that the relationship between LM and petiole mass (PM) may be evolutionarily stable because too much biomass investment in petiole will reduce the resource available to lamina development (e.g., small LA), which makes against leaf light acquisition, whereas too much investment in lamina will increase the risk of leaves falling prematurely due to a lack of sufficient support (Yamada et al., 1999; Niinemets et al., 2006; Li et al., 2008; Yoshinaka et al., 2018). The relationship, however, was found positively allometrical (i.e., higher mass variation in petiole than lamina with increasing leaf mass) in several studies (Niinemets and Kull, 1999; Niinemets et al., 2006; Levionnois et al., 2020), in which the authors assumed (without empirical evidence) that large-sized leaves had to invest more in support structures than small-sized leaves because the former experienced large static loads on the petiole and lamina (caused by long bending and torsional moments on petiole) and extra dynamic loads of drag forces on lamina surface (due to large lamina stress area; Niinemets and Kull, 1999; Li et al., 2008; Bal et al., 2011; Fan et al., 2017). Thus, given that the assumption is correct, we can suppose that the allometric PM–LM relationship is caused mainly by LA, whereby a positive PM–LA relationship is expected after controlling for the effect of PM, especially in stressful alpine (AL) environments where leaves undergo stronger natural drag forces (wind blowing, snow covering, air–moisture freezing, etc.) on the lamina surface (Anten et al., 2010; Louf et al., 2018).

In brief, the petiole–lamina relationship in different dimensions (length, area, and mass) can represent different aspects of plant functional tradeoff or adaptation to environmental gradients (Niinemets, 1998; Li et al., 2008; Levionnois et al., 2020), and thus, it should be studied together. However, the current studies, often based on intraspecific comparison of a single species or interspecific comparison of a small number of species (Niinemets and Kull, 1999; Yamada et al., 1999; Niinemets and Fleck, 2002; Anten et al., 2010; Ray and Jones, 2018; Levionnois et al., 2020), have paid attention mostly to one or two dimensions of the relationship along a small environmental gradient (Zhong et al., 2019). As a result, these studies often produce conflicting findings and can hardly provide a reliable assessment of the relationship. In the study reported here, using a leaf trait database of 537 populations of 325 common woody species from various climate or vegetative zones representing a near 2,000-m gradient of elevation in the eastern part of the Qinghai-Tibetan Plateau (QTP), we present the first comprehensive investigation of the petiole–lamina relationship of an entire woody flora in all three dimensions (length, area, and mass). Specifically, the relationship was examined across species and within each zone. We also analyzed its allometric or isometric pattern and compared the difference of the relationship with and without controlling for related traits. The specific objectives and the associated hypotheses of this study are presented in Table 1.

**Table 1:** The main topics and hypotheses dealt with in this study.

**Relationship**	**PL-LL**	**PCA-LA**	**PM-LM**
**(a)**			
Isometric (RC = 1)	Showing a balance between adaptive light interception and support costs of petiole growth; expected to be common in moderate environments with relatively sparse vegetation cover	Showing a balance between enhancing support/protection costs and nutrient/water transport in petiole growth; common in moderate environments	Petioles support mainly the static load of leaf mass, common in moderate environments without significant natural drag forces (e.g., wind) on lamina surface
Positively allometric (RC > 1)	Showing that petiole growth is influenced by a higher demand for light interception; common in dense vegetation with obvious vertical layers	Showing a higher demand for enhancing support/protection costs in petiole growth; common in stress environments	Petioles support both the static and dynamic loads by drag forces; common in stress environments with strong natural forces
Negatively allometric (RC < 1)	Showing that petiole development is influenced by a higher demand for support costs; common in sparse vegetation or stress environments	Showing a higher demand for enhancing nutrient/water transport in petiole growth; common in hot and/or humid environments	Other mechanisms of reducing a petiole support requirement for static leaf loads with increasing leaf mass
**Relationship**	**PL–R**_**LA/LL**_ **(PL–LA relationship after controlling for LL)**	**PCA–R**_**LM/LA**_ **(PCA–LM relationship after controlling for LA)**	**PM–R**_**LA/LM**_ **(PM–LA relationship after controlling for LM)**
**(b)** Non-significant (or positive)	The effect of LA on PL can (or cannot) be explained fully by LL, representing no (or significant) additional influence of leaf width or leaf shape on petiole length	The effect of LM on PCA can (or cannot) be explained fully by LA, representing no (or significant) additional influence of lamina structure on petiole structure	The effect of LA on PM can (or cannot) be explained fully by LM, representing no (or significant) additional static gravity or dynamic drag force caused by lamina size variation on biomass allocation to petiole

*RC, regression coefficients; PL, petiole length; LL, lamina length; PCA, petiole crosssectional area; LA, lamina area; PM, petiole mass; LM, lamina mass*.

## Materials and Methods

### Study Area

The study region is located on the east edge of QTP (101°05′-104°20′ E, 33°25′-35°30′ N, about 40,000 km^2^) where the altitude is the strongest determinant of bioclimatic gradients. In the region, within only 160 km one can move from warm-temperate to AL zone. Woody vegetation types and their climate characteristics also differ greatly among altitudes (see below and Table 2).

**Table 2:** The meteorological parameters at each sampling altitudinal transect are the approximate range of climate change for many years in the study area.

**Altitude (m)**	**Climatic zone**	**Zonal woody vegetation type**	**MAT (°C)**	**FFM**	**LGS (days)**	**Canopy height (m)**
1,800	Warm-temperate	Deciduous-evergreen broadleaf forest	10–13	7–8	>250	15–18
1,900	Warm-temperate	Deciduous-evergreen broadleaf forest	9–12	6–7	230–250	12–15
2,000	Warm-temperate	Deciduous broadleaf forest	8–11	ca. 6	220–240	12–15
2,100	Warm-temperate	Deciduous broadleaf forest	7–10	5–6	210–240	10–15
2,200	Cool-temperate	Deciduous broadleaf forest	6–9	ca. 5	210–230	10–12
2,300	Cool-temperate	Deciduous broadleaf forest	6–8	4–5	200–230	10–12
2,400	Cool-temperate	Deciduous broadleaf forest	5–7	ca. 4	200–220	8–12
2,500	Cool-temperate	Deciduous broadleaf-conifer forest	4–7	3–4	190–220	8–10
2,600	Cool-temperate	Deciduous broadleaf-conifer forest	3–6	3–4	180–210	7–10
2,700	Subalpine	Deciduous broadleaf-conifer forest	3–5	ca. 3	180–200	6–10
2,900	Subalpine	Mixed forest-deciduous shrub	1–4	2–3	160–180	5–8
3,100	Subalpine	Mixed forest-deciduous shrub	0–3	1–2	150–170	3–6
3,300	Subalpine	Mixed forest-deciduous shrub	−1 to 1	0–1	140–160	2–5
3,500	Alpine	Deciduous-evergreen broadleaf shrub	−2 to 0	0	130–150	1–2.5
3,700	Alpine	Deciduous-evergreen broadleaf shrub	−3 to −1	0	120–140	0.5–1.5

*Climate data was downloaded from National Meteorological Information Center (http://data.cma.cn/). “Mixed forest” means mixed conifer–deciduous broadleaf forest. Vegetation type before and after minus (–) sign represent locally dominant and secondary vegetation, respectively. The frost-free months (FFM) refer to the absolute frost-free months (periods). The length of the growing season (LGS) is based on the average phenological performance of the local dominant woody species. MAT, mean annual temperature*.

### Plant Sampling

**Figure 1:**
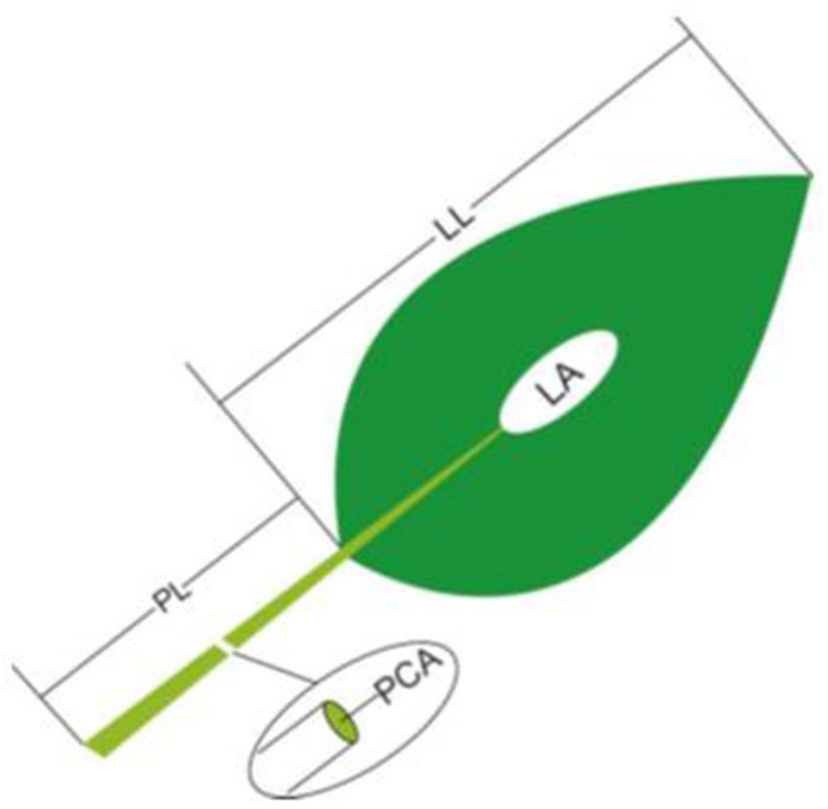
Schematic representation of the method of leaf trait measurement. PL, petiole length; LL, lamina length; PCA, petiole crosssectional area; LA, lamina area.

In summer and early autumn of 2018 and 2019, we set 15 altitudinal transects located at each 100-m (below 2,700 m a.s.l.) or 200-m (above 2,700 m a.s.l. for less woody species, smaller woody vegetation area and lower altitudinal difference in species composition) interval (Table 2). At each transect, two to four 300 m ^*^ 300 m sites (altogether 43 sites) were sampled to avoid possible sampling bias. These transects can be divided into four climatic zones, including warm-temperate (WT), cool-temperate (CT), subalpine (SA), and AL, or into five vegetation types, including deciduous-evergreen broadleaf forest (DEBF), deciduous broadleaf forest (DBF), deciduous broadleaf-conifer forest (DBCF), mixed forest-deciduous shrub (MFDS), and deciduous-evergreen broadleaf shrubs (DEBS). At every site, leaf materials were gathered from each dominant as well as common species. For leaf materials of the same species (seen in Supplementary Data), different populations were treated as one sample in the same transect but as different samples in different transects. The total number of samples (populations) in all transects was 537, belonging to 325 species in 95 genera of 46 families (based on the Angiosperm Phylogeny Group IV classification system, undated in 2016). For each sample, three to five individuals that grew well (i.e., mature and living in the habitat with sufficient nutrition and low disturbance) were randomly selected. Then, for each individual, two to three branches with five to 20 mature, healthy, fully expanded, and undamaged leaves on each branch were chosen at random at the outer canopy to avoid obvious differences in light conditions. Ninety populations of 56 species had compound leaves (Supplementary Data); for these, the whole leaves, rather than the leaflets, were measured. For each sample, each leaf was divided into petiole and lamina, and then the following traits were calculated as:

(1) Petiole cross-sectional area: For each sample, at least 10 leaves were chosen from different individuals to measure petiole diameter (PD), the diameter of the middle position from lamina base to the end of the petiole, by using a spiral-micrometer, and PCA was calculated as: PCA = π^*^(PD/2)^2^.
(2) The LL, LA and PL: For each sample, five to 20 leaves (or two to four large leaves) from different individuals were bulked together representing one replicate. LA was determined by scanning the leaves with a flatbed scanner (HP LaserJet 1320) in three to four repetitions and then analyzing the pictures with image analysis software (Image J; http://rsb.info.nih.gov/ij). In each replicate, all selected leaves were carefully placed on the scanner to avoid overlap and fully expand bent or contracted leaves. LL and PL were determined by analyzing scanned pictures (eight to 15 leaves were randomly selected for each sample; Figure 1).
(3) The LM and PM: All the imaged leaves, including the lamina and petiole, were dried at 70°C to a constant mass and weighed to the nearest 0.0001 g, respectively. Then, the obtained dry mass was divided by leaf number to calculate the individual LM and PM.

### Statistical Analysis

Data on leaf traits were log-transformed before analyses to fit a normal distribution. The petiole–lamina relationship was analyzed by using both crossspecies analysis and metaanalysis, whereby petiole traits were treated as dependent variables in all binary relationship analyses. All analyses were performed in R 3.6.1.

#### Crossspecies Analysis

In the analyses, leaf traits of different samples for the same species were averaged; altogether, 325 species were used. Specifically, we performed linear regression on each of three dimensions of the petiole–lamina relationships (PL–LL, PCA–LA, and PM–LM) for all species, simple-leaf species, and compound-leaf species. The heterogeneity of the relationship slopes and the intercepts among simple-leaf, compound-leaf, and all species were tested by ANCOVA.

#### Meta-Analysis

We examined six sets of petiole–lamina relationships (PL–LL, PCA–LA, PM–LM, PL–R_LA/LL_, PCA–R_LM/LA_, and PM–R_LA/LM_) in each altitudinal transects, with R_LA/LL_, R_LM/LA_, and R_LA/LM_ being the residuals of LA on LL, LM on LA, and LA on LM, respectively. Thus, the PL–R_LA/LL_, PCA–R_LM/LA_, and PM–R_LA/LM_ relationships will help assess whether the associations of PL–LL, PCA–LA, and PM–LM are independent of the variation in LA, LM, and LA, respectively. Effect sizes for each set of petiole–lamina relationship were analyzed by the “Metafor” package. We used meta-analysis models to calculate the mean effect size across transects by weighting each transect-specific effect size by its corresponding standard error. The mean effect size across altitudinal transects and for each climate zone or vegetation type was calculated by applying the random effects model, and its 95% confidence intervals (95% CI) were calculated by bootstrapping with 4,999 iterations. We used between-group heterogeneity (*Q*_between_) to determine the differences in effect size between different altitudinal transects and tested its significance based on the critical value in a standard chi-square table (Zvereva et al., 2010).

## Results

### Crossspecies Pattern

Across all species, the three dimensions of the petiole–lamina relationships were all significantly positive, with the regression slopes being non-significantly >1, significantly smaller than 1, and significantly >1 for the PL–LL, PCA–LA, and PM–LM relationships, respectively. The simple-leaf species showed the slopes and intercepts similar to those of all species in the three dimensions of the petiole–lamina relationships, but the compound-leaf species showed a significantly lower slope in the PL–LL relationship and a higher intercept in the PL–LL and PM–LM relationships compared with those of all species (Table 3).

**Table 3:** Summary of three dimensions of linear relationship (PL–LL, PCA–LA, and PM–LM) for all, simple-leaf and compound-leaf species group, respectively.

**Relationship**	**All species**		**Simple-leaf species**		**Compound-leaf species**		**Comparison**	
	**Slope (B_**A**_)**	**Intercept (A_**A**_)**	**Slope (B_**S**_)**	**Intercept (A_**S**_)**	**Slope (B_**C**_)**	**Intercept (A_**C**_)**	
PL–LL	1.080 ± 0.146	−1.693 ± 0.288	1.134 ± 0.183	−1.862 ± 0.348	0.800 ± 0.217	−0.757 ± 0.493	B_S_=B_A_>B_C_, A_C_>A_A_=A_S_
PCA–LA	0.738 ± 0.046	−6.070 ± 0.343	0.762 ± 0.053	−6.198 ± 0.392	0.739 ± 0.096	−6.290 ± 0.772	B_S_=B_C_=B_A_, A_A_=A_S_=A_C_
PM–LM	1.193 ± 0.061	−2.364 ± 0.159	1.150 ± 0.065	−2.593 ± 0.171	1.139 ± 0.119	−1.829 ± 0.284	B_A_=B_S_=B_C_, A_C_>A_A_≧A_S_

*For each linear relationship, the mean ± 95%CI (confidence interval) of the slope (B) and intercept (A) are shown. The “Comparison” column represented the difference in slope or intercept (“>”, “≧” and “=” were significant difference at P < 0.01, P < 0.05, and P > 0.05, respectively) among species groups. PL, petiole length; LL, lamina length; PCA, petiole cross-sectional area; LA, lamina area; PM, petiole mass; LM, lamina mass; B_A_, B_S_, and B_C_: the mean ± 95%CI (confidence interval) of the slope of the linear relationship for all species, simple-leaf species and compound-leaf species; A_A_, A_S_ and A_C_: the mean ± 95%CI of the intercept of the linear relationship for all species, simple-leaf species and compound-leaf species*.

### Patterns in Different Transects, Climate Zones, and Vegetation Types

#### The PL–LL, PCA–LA, and PM–LM Relationship

**Figure 2:**
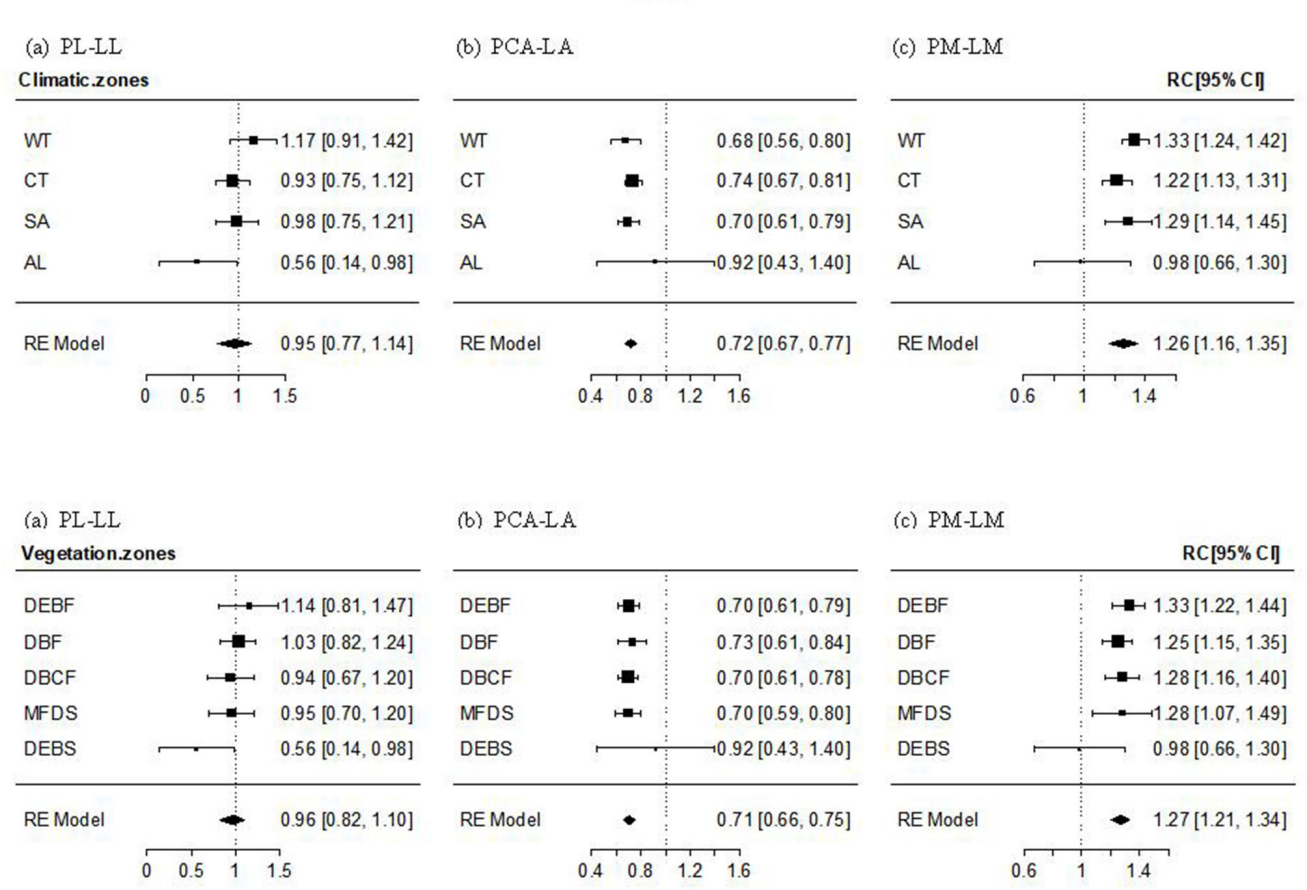
Mean effect size and 95% intervals of the relationship between petiole length and lamina length (PL–LL, **a**), between petiole crosssectional area [PCA and lamina area (PCA–LA, **b**)], and between petiole mass and lamina mass (PM–LM, **c**) for different climate zones and vegetation types. WT, warm-temperate; CT, cool-temperate; SA, subalpine; AL, alpine; DEBF, deciduous-evergreen broadleaf forest; DBF, deciduous broadleaf forest; DBCF, deciduous broadleaf-conifer forest; MFDS, mixed forest-deciduous shrub; DEBS, deciduous-evergreen broadleaf shrub.

The PL–LL relationship was significantly positive in almost all transects, climate zones, and vegetation types, with the mean effect size being non-significantly different from 1. The effect size of the relationship decreased gradually (but non-significantly; Q_between_ = 14.204, *P* = 0.435) with altitude, from non-significantly >1 in WT forest, to approximately 1 in cold-temperate and SA forests and shrubs, and significantly smaller than 1 in AL shrubs (Figure 2a, Supplementary Figure 1a). The mean effect size of PCA–LA relationship was significantly positive, but smaller than 1. The effect size of the relationship showed a non-significant increase with an increase in altitude (Q_between_ = 19.029, *P* = 0.164), being significantly smaller than 1 in temperate and SA forests or shrubs, but non-significantly different from 1 in AL shrubs (Figure 2b, Supplementary Figure 1b). The PM–LM relationship was also significantly positive in most transects, climate zones, and vegetation types, with the mean effect size being significantly >1. The altitudinal trend in the effect size was non-significantly negative (Q_between_ = 14.015, *P* = 0.449), being significantly >1 in temperate and SA forests or shrubs, but non-significantly different from 1 in AL shrubs (Figure 2c, Supplementary Figure 1c).

#### The PL–R_LA/LL_, PCA–R_LM/LA_, and PM–R_LA/LM_ Relationships

**Figure 3:**
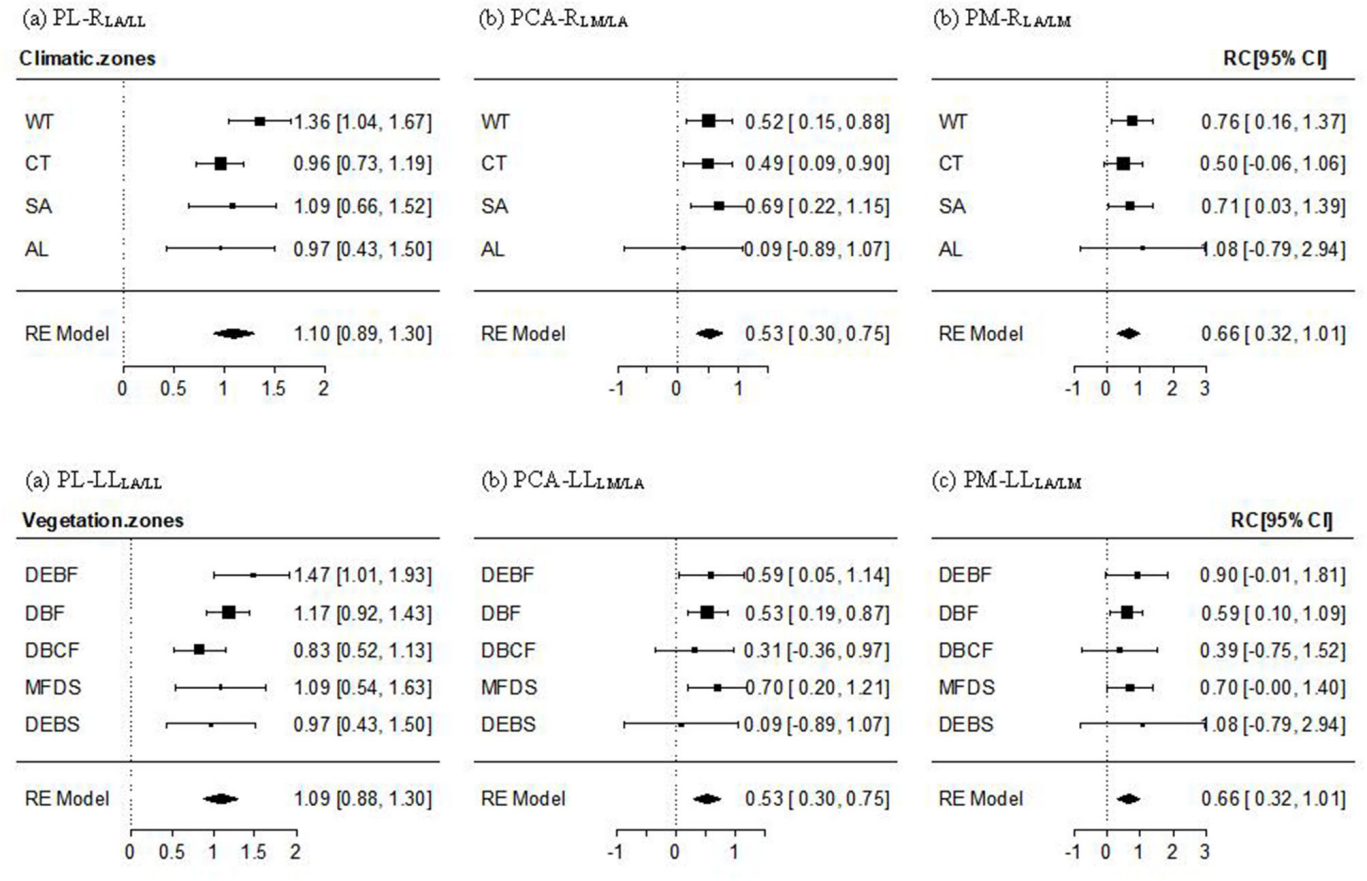
Mean effect size and 95% confidence intervals of the PL–R_LA/LL_ **(a)**, PCA–R_LM/LA_ **(b)**, and PM–R_LA/LM_ **(c)** relationship for different climate and vegetation zones. Abbreviations of leaf traits, climate zones, and vegetation types were as specified in Figure 2. R_LA/LL_, R_LM/LA_, and R_LA/LM_ were the regression residuals of LA on LL, LM on LA, and LA on LM, respectively.

For the PL–R_LA/LL_ relationship, a non-significant altitudinal pattern was found (Q_between_ = 9.389, *P* = 0.805), whereby the mean effect size across all transects and the effect size for most climate zones and vegetation types, except for the lowest-altitude WT and DEBF zones, were significantly positive but non-significantly different from 1 (Figure 3a, Supplementary Figure 2a). The PCA-R_LM/LA_ relationship was significantly positive with the mean effect size being near 1/2. An altitudinal pattern in the relationship varied from significantly positive in low-altitude forests (WT, CT, DEBF, and DBF), to significantly or non-significantly positive in mid altitude forests, to being approximately 0 in AL shrubs (Figure 3b), Supplementary Figure 2b). The mean effect size of the PM–R_LA/LM_ relationship was also significantly positive, with a value being near 2/3. The altitudinal difference in the effect size was non-significant (Q_between_ = 1.369, *P* = 0.999), with a slightly lower value in the mid altitude vegetation types (CT and DBCF; Figure 3c, Supplementary Figure 2c).

## Discussion

We showed a different pattern of three dimensions of the petiole–lamina relationships. These relationships were also different between leaf forms, altitude transects, climate zones, and vegetation types. Moreover, we found significant additional influences of LA, LM, and LA on the PL–LL, PCA–LA, and PM–LM relationship (Figure 3, Supplementary Figure 2), respectively. These findings imply that multiple mechanisms may operate simultaneously in governing petiole/lamina development and their relationship. Below, we discuss our findings and also potential explanations for some of the more unexpected results.

On the one-dimension scale, PL was found to be positively correlated with LL (Table 3, Figure 2), supporting our hypothesis that long-leaf species may experience obvious leaf overlapping and benefit more from elongating petiole in enhancing light interception despite the additional costs in leaf support (King and Maindonald, 1999; Niklas, 1999; Poorter and Rozendaal, 2008; Vogel, 2009; Roig-Villanova and Martíinez-Garcíia, 2016). The PL–LL relationship was isometric within most transects or zones (Figure 2, Supplementary Figure 1), showing a proportional length variation in petiole and lamina. This result suggests that an overall balance between adaptive light interception vs. support costs in petiole growth may be universal in many environments and a stable mechanism for most woody species, regardless of different leaf sizes and shapes. Moreover, we found a reduction in effect size of the relationship with increasing altitude, varying from non-significantly >1 at the lowest altitude to significantly lower than 1 at the highest altitude (Figure 2, Supplementary Figure 1). The result may imply that the balance shifts toward increasing PL to maximize light interception in WT forests with a tall and dense canopy and obvious vertical stratification, but toward decreasing PL in AL shrubs to provide stronger support to resist strong winds (Niklas, 1999; Niinemets et al., 2007; Vogel, 2009; Anten et al., 2010; Louf et al., 2018) and also to reduce excessive leaf exposure to high ultraviolet (UV) radiation (Castro-Díez et al., 2000; Li et al., 2019). Also, our results showed a significant positive PL–R_LA/LL_ relationship within most altitudes or zones (Figure 3, Supplementary Figure 2), supporting the hypothesis of a significant additional effect of lamina width and lamina shape on the variation in PL (Table 1). To our surprise, the PL–R_LA/LL_ relationship was isometric, which was the same as the pattern in the PL–LL relationship, suggesting that lamina growth in two directions (parallel and vertical to petiole) has equal effects on the growth and development of petiole. A lack of significant difference in the PL–R_LA/LL_ relationship among climate zones or vegetation types implies that the equal effect of lamina growth in two directions on PL may be the result of evolutionary tradeoffs among leaf traits, leading to less dependence of the relationship on the environmental variations.

In contrast, petiole crosssectional area was negatively isometrically scaled to (i.e., increased disproportionately with) LA (Figure 2, Table 3), which is in contrast to the “pipe-model theory” that an isometric relationship is expected (Niklas, 1992; Ray and Jones, 2018; Levionnois et al., 2020). There are two possible explanations for this result. Firstly, the number of cell layers in some structures in petioles, such as epidermis and cambium, is relatively fixed and generally varies disproportionately with vascular structure (Song and Hong, 2018), resulting in a smaller variation in PCA than LA. Secondly, xylem in petiole is composed of vessels of different sizes. According to the Hagen–Poiseuille law in the hydraulic conductivity of the conducting tissues (Niklas et al., 2009; Gebauer et al., 2019), narrow vessels in a large density often occupy a larger area of secondary xylem than large vessels in small density, but small-density larger vessels, due to their lower water transport resistance, tend to transport a high amount of water than large-density narrow vessels (Sack and Frole, 2006; Lintunen and Kalliokoski, 2010; Gebauer et al., 2016). Thus, petioles of large leaves generally develop a small amount of large vessels, rather than a large number of small ones, although they may have similar total petiole xylem cross section area, to respond to an increased requirement for water transport, which would weaken the dependence of crosssectional area of the vascular structure of the petiole on the LA. This is supported by a few studies on LA varying isometrically with the maximum diameter of the xylem vessels (*D*_max_), rather than with the number or density of vessels (Gleason et al., 2018; Zhong et al., 2019; Levionnois et al., 2020). Moreover, we have shown a significant and large effect size for the PCA–LA relationship in AL shrubs, whose reason may be that a high percentage of supportive/protective tissues (e.g., collenchyma or sclerenchyma cells) or an increase in storage tissues (e.g., parenchyma cells) is needed in the petioles of high-altitude species to adapt to increasing stresses (Li et al., 2008; Anten et al., 2010; Pan et al., 2013; Gleason et al., 2018).

Generally, PM was positively allometrically scaled to lamina mass, suggesting that the proportion of investment in the petiole increases with an increase in leaf mass. The result is in accordance with the most frequently reported findings (Niinemets and Kull, 1999; Niinemets et al., 2006, 2007; Li et al., 2008; Fan et al., 2017; Levionnois et al., 2020), but supports neither the explanation in these studies nor our hypothesis (Table 1) for the allometric relationship based on to the altitudinal reduction in the effect size of the relationship. If the positive allometry arises mainly from petioles needing to support extra dynamic loads caused by drag forces, the effect size should increase with altitude to adapt to the increasing natural forces on the lamina surface. The difference in community structure and climate between altitudinal transects may contribute to the results. Firstly, for temperate and SA forests with closed and tall canopy, strong light competition and long-distance nutrient and water transport between root and canopy may force the large-leaf species to invest a high proportion of biomass to petiole for (i) increasing leaf light interception (by elongating petiole length) and (ii) maintaining a high rate of photosynthesis and transpiration (by increasing the number and/or crosssectional surface area of the xylem/phloem vessels) (Bell and Galloway, 2007; Sarlikioti et al., 2011; Rosell et al., 2017). In contrast, AL shrubs benefit from a smaller increase in PM than LM because a low investment of mass in the petiole, associated with short petiole and low vessel density, can reduce leaf damage due to excessive solar radiation and low leaf temperature caused by high transpiration rate (Vogel, 2009; Anten et al., 2010; Peng et al., 2015). Our results, therefore, illustrate the importance of plant competition for light and the photosynthesis/transpiration strategy in determining a leaf biomass allocation pattern in woody perennials (Sarlikioti et al., 2011).

The mean effect size of the PCA–R_LM/LA_ and PM–R_LA/LM_ relationship were both significantly positive (i.e., >0; Figure 3, Supplementary Figure 2), suggesting significant additional influences of LM and LA on the PCA–LA and PM–LM relationship. The positive PCA–R_LM/LA_ relationship matches well our hypothesis in Table 1 that the variation in lamina structure (e.g., the number of cell layers in the palisade and sponge tissues or the proportion of protective tissue) can exert a strong influence on the variation in petiole structure, such as the number and proportion of vascular, protective, and supportive tissue components; whereas the positive PM–R_LA/LM_ relationship can be explained by the hypothesis (in Table 1) that the variation in LA, reflecting the changes in the static loads of gravity force on petiole caused by the variation in the bending and torsional moments, and/or the change in the dynamic drag force on petiole caused by the variation in stress area of lamina surface, can affect leaf biomass allocation to petiole (Niklas, 1999; Niinemets et al., 2006; Sack and Frole, 2006; Li et al., 2008; Anten et al., 2010; Bal et al., 2011; Gebauer et al., 2016; Louf et al., 2018).

Compared with our meta-analysis whose mean effect size (E) showed the within-site (local) pattern of petiole–lamina relationship, the crossspecies analysis exhibited mainly a regional among-site pattern of the relationship based on its regression slope (B). In the study, a miniaturized lamina (low LL, LA, and LM) was found to be one of the most significant among-site leaf variations in woody plants at high altitude (the altitudinal variation in leaf traits was presented in Supplementary Table 1). Therefore, our finding of, a higher crossspecies PL–LL relationship (i.e., higher B than E), may be attributed to an increase in the demand for petiole elongation in most low-altitude leaves (often with long lamina), whereas a lower crossspecies PM–LM relationship may be associated with an increase in biomass allocation to petiole for most high-altitude leaves (often with small lamina mass). However, because of the lack of leaf anatomical data, we cannot determine that the higher biomass allocation to petiole at high altitude is due to its higher demand for support/protection (i.e., more supportive/protective tissues in petiole), or resource storage (i.e., more storage tissues in petiole), or both.

We did not find any significant difference in the slope of the PCA–LA and PM–LM relationship between the simple-leaf and compound-leaf species (Table 3), implying that the variation in a leaf tradeoff between the support/protection costs and the nutrient/water transport, or in a leaf biomass allocation strategy is independent of the leaf form. In contrast, compound leaves showed a significantly lower slope of the PL–LL relationship than simple leaves (Table 3). The reason may be that a compound leaf is composed of leaflets, petiole, and petiolules. The petiolules can extend the length and width of a compound leaf, which may help diminish self-shading among leaves (or leaflets) and increase leaf light interception (Niinemets, 1998; Niinemets et al., 2006; Bell and Galloway, 2007; McCulloh et al., 2009; Xu et al., 2009; Pan et al., 2013), thereby reducing the dependence of PL variation on LL variation (Li et al., 2008). The extension of the length and width of compound leaves, however, leads to a high static load and a dynamic drag force on the petiole due to the increase in the petiole bending/torsional moments and the lamina stress area. This necessitates a high leaf biomass allocation to petiole and contributes to our result of a higher intercept of the PM–LM relationship for the compound leaves than the simple leaves.

In summary, our study illustrates the importance of plant functional tradeoffs, especially the tradeoff between adaptive light interception and leaf support costs, between enhancing nutrient, water transport, and the support/protection costs, and between increasing leaf photosynthetic efficiency or transpiration rate and leaf protection against high solar radiation or low temperature; meanwhile, we provide evidence of a shift in the tradeoffs determining leaf growth and variation along the environmental gradients. In addition, different dimensions of the petiole-lamina relationship, reflecting different aspects of plant functional tradeoffs, should be considered together in examining the regional distribution and local adaptation of perennial plant species.

## Data Availability Statement

The original contributions presented in the study are included in the article/Supplementary Material, further inquiries can be directed to the corresponding author/s.

## Author Contributions

WQ conceived the ideas. YL and XK analyzed the data. YL, XK, and WQ contributed to the writing of the manuscript. All the authors collected the data, read, commented on, and approved this version of the manuscript.

## Funding

The study was supported by the Project of the National Natural Science Foundation of China (31770448, 31870411, and 32171518), the National Key Research and Development Program of China (Grant No. 2017YFC0504801), and the Science and Technology Support Project of Ecological Grassland Restoration and Management in Gansu Province granted to WQ (2020-72). The study is also supported by Gannan Grassland Ecosystem National Observation and Research Station.

## Conflict of Interest

The authors declare that the research was conducted in the absence of any commercial or financial relationships that could be construed as a potential conflict of interest.

## Publisher's Note

All claims expressed in this article are solely those of the authors and do not necessarily represent those of their affiliated organizations, or those of the publisher, the editors and the reviewers. Any product that may be evaluated in this article, or claim that may be made by its manufacturer, is not guaranteed or endorsed by the publisher.

## Supplementary Material

Supplementary Figure 1 Effect size and 95% confidence intervals of the PL–LL **(a)**, PCA–LA **(b)**, and PM–LM **(c)** relationship for different altitudinal transects. Abbreviations of leaf traits were as specified in Figure 2.

Supplementary Figure 2 Effect size and 95% confidence intervals of the PL–R_LA/LL_ **(a)**, PCA–R_LM/LA_ **(b)**, and PM–R_LA/LM_ **(c)** relationship for different altitudinal transects. Abbreviations of leaf traits were as specified in Figures 2, 3.

Supplementary Table 1 Mean± standard error of six petiole and lamina traits for 15 altitudinal transects.

Supplementary Data A comparison of six petiole and lamina traits for 325 woody species (537 populations). PL, petiole length (cm); LL, lamina length (cm); PCA, petiole cross-sectional area (mm^2^); LA, lamina area (mm^2^); PM, petiole mass (g); LM, lamina mass (g).
