# Supplementary figures and images for: Geographic Variation in the Petiole–Lamina Relationship of 325 Eastern Qinghai–Tibetan Woody Species: Analysis in Three Dimensions
Source: Front Plant Sci. 2021 Oct 28;12:748125. doi: 10.3389/fpls.2021.748125 (PMC8583490; doi:10.3389/fpls.2021.748125)

**Fig. S1**

**(a) PL-LL**

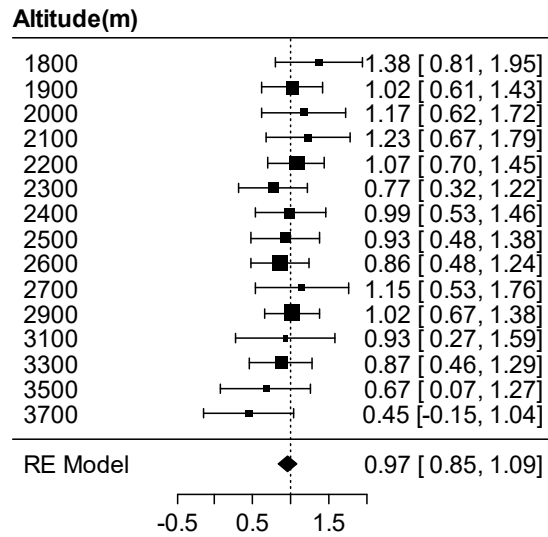

**(b) PCA-LA**

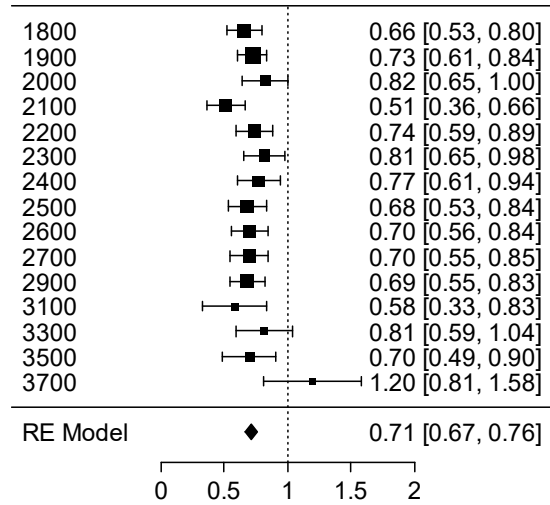

**(c) PM-LM**

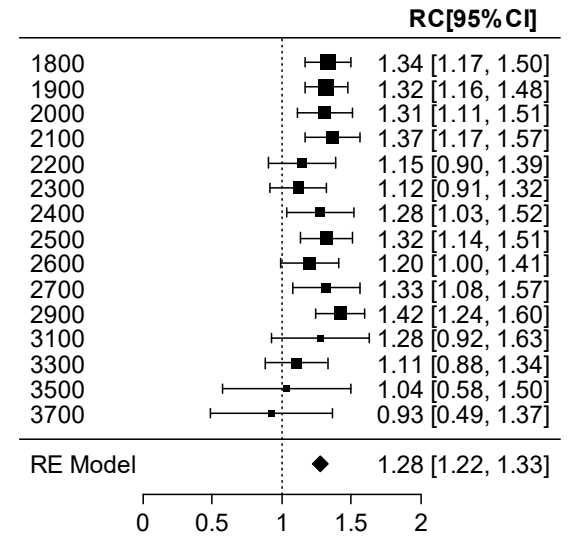

Supplement: Supplementary Figure 1 — Effect size and 95% confidence intervals of the PL–LL (a), PCA–LA (b), and PM–LM (c) relationship for different altitudinal transects. Abbreviations of leaf traits were as specified in Figure 2. [file Image_1.pdf]

Fig. S2

(a) PL-LL<sub>LA/LL</sub>

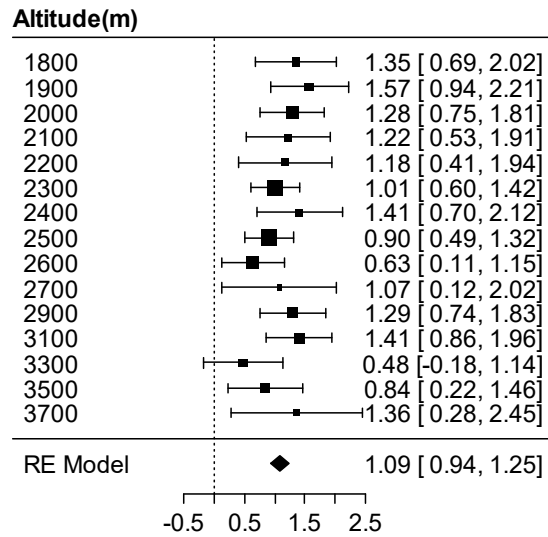

(b) PCA-LL<sub>LM/LA</sub>

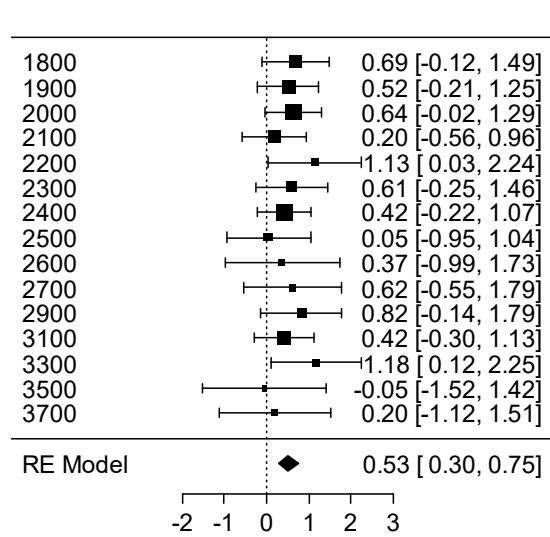

(c) PM-LL<sub>LA/LM</sub>

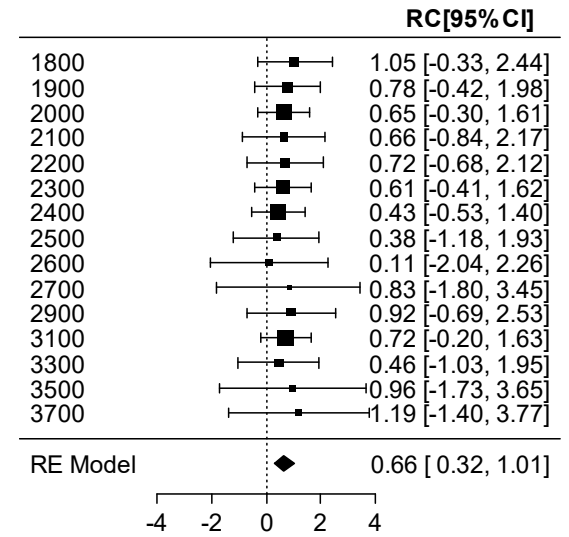

Supplement: Supplementary Figure 2 — Effect size and 95% confidence intervals of the PL–RLA/LL (a), PCA–RLM/LA (b), and PM–RLA/LM (c) relationship for different altitudinal transects. Abbreviations of leaf traits were as specified in Figures 2, 3. [file Image_2.pdf]
